# Strongly Bactericidal All-Oral β-Lactam Combinations for the Treatment of Mycobacterium abscessus Lung Disease

**DOI:** 10.1128/aac.00790-22

**Published:** 2022-09-01

**Authors:** Dereje A. Negatu, Matthew D. Zimmerman, Véronique Dartois, Thomas Dick

**Affiliations:** a Center for Discovery and Innovation, Hackensack Meridian Health, Nutley, New Jersey, USA; b Center for Innovative Drug Development and Therapeutic Trials for Africa (CDT-Africa), Addis Ababa University, Addis Ababa, Ethiopia; c Department of Medical Sciences, Hackensack Meridian School of Medicine, Nutley, New Jersey, USA; d Department of Microbiology and Immunology, Georgetown University, Washington, DC, USA

**Keywords:** nontuberculous mycobacteria, NTM, synergy, sulopenem, tebipenem, cefuroxime, amoxicillin, avibactam

## Abstract

Bioactive forms of oral β-lactams were screened *in vitro* against Mycobacterium abscessus with and without the bioactive form of the oral β-lactamase inhibitor avibactam ARX1796. Sulopenem was equally active without avibactam, while tebipenem, cefuroxime, and amoxicillin required avibactam for optimal activity. Systematic pairwise combination of the four β-lactams revealed strong bactericidal synergy for each of sulopenem, tebipenem, and cefuroxime combined with amoxicillin in the presence of avibactam. These all-oral β-lactam combinations warrant clinical evaluation.

## TEXT

Mycobacterium abscessus lung disease is treated with an oral macrolide (clarithromycin [CLR] or azithromycin) in combination with several largely underperforming antibiotics, including parenteral amikacin; one of the two parenteral β-lactams, imipenem (IPM) or cefoxitin (FOX); and tigecycline (1). Patients are often treated for years until sputum cultures remain negative for 12 months if culture conversion is achieved. Chemotherapies, complicated by the need to use injectable drugs, are not only long but often toxic (1, 2). Treatment is further tangled by widespread inducible resistance to the oral macrolide component due to the presence of the ribosome methylase gene *erm41*, particularly in M. abscessus subsp. *abscessus* (3–6). In short, there is no reliable cure for M. abscessus lung disease. Novel, well-tolerated, bactericidal, and, importantly, oral treatment options are sorely needed (7, 8).

β-Lactams are bactericidal and display overall excellent tolerability profiles (9). However, IPM and FOX, the standard-of-care carbapenem and cephalosporin, respectively, are administered intravenously, limiting their clinical utility given the very long treatment duration required to control or cure M. abscessus lung disease. They also show modest *in vitro* activity (10, 11), leading to poor pharmacokinetic-pharmacodynamic target attainment compared to those achieved against other bacterial infections.

Different classes of β-lactams, and different members within a class, differentially inhibit the numerous M. abscessus transpeptidases and other enzymes involved in peptidoglycan synthesis (12–17). Thus, multiple recent reports have demonstrated the potential of combining two β-lactams to achieve additive and synergistic effects *in vitro* (18–22) as well as *in vivo* (23).

A number of oral β-lactams are in clinical development or in clinical use for other bacterial infections (24, 25). Furthermore, an oral form (ARX1796) of the β-lactamase inhibitor avibactam (AVI), inhibiting the major β-lactamase MAB_2875 of M. abscessus (10, 26), recently entered clinical development (27) (ClinicalTrials.gov identifier NCT03931876).

**FIG 1:**
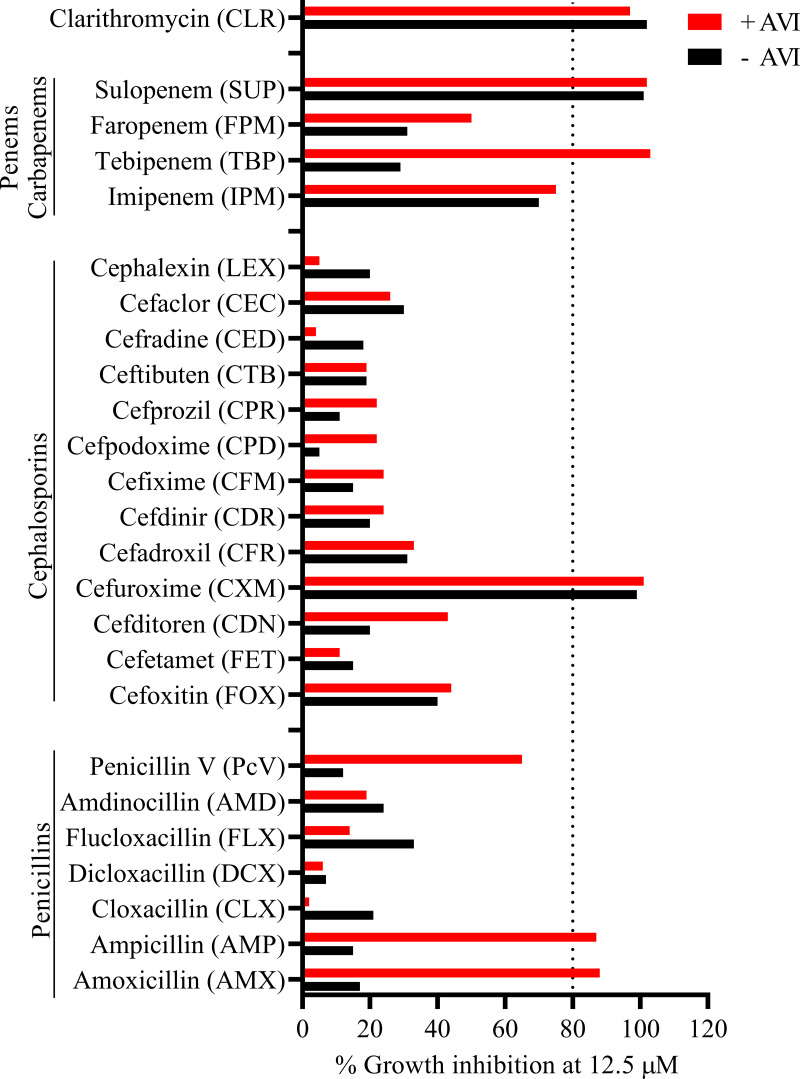
Single-point growth inhibition screen of β-lactams with and without 4 μg/mL AVI against M. abscessus ATCC 19977. A collection of the bioactive forms of 22 oral β-lactams was screened at 12.5 μM. Percent growth inhibition is shown. Dashed line, 80% growth inhibition. CLR was included as positive control IPM and FOX as clinically used parenteral comparators. The experiment was carried out twice, yielding similar results. Compound sources, oral prodrug forms (if applicable), and clinical status are described in Table S1 in the supplemental material.

Here, our goal was to identify oral β-lactam pairs that exert synergistic bactericidal activity (with or without oral AVI) and can be repurposed to treat M. abscessus lung disease. First, a collection of the bioactive forms of 22 oral β-lactams, including penems, carbapenems, cephalosporins, and penicillins, was screened at a single concentration of 12.5 μM with and without the bioactive form of AVI at a fixed concentration of 4 μg/mL (14 μM) to identify antibiotics with attractive anti-M. abscessus growth inhibitory activity (28). Growth inhibition against the type strain M. abscessus subsp. *abscessus* ATCC 19977 was measured in 7H9 broth using optical density at 600 nm (OD_600_) as readout (29). Applying >80% growth inhibition as cutoff, we identified one penem (sulopenem [SUP]), one carbapenem (tebipenem [TBP]), one cephalosporin (cefuroxime [CXM]), and two penicillins (ampicillin [AMP] and amoxicillin [AMX]) as active agents. At 12.5 μM, SUP and CXM were equally active with and without AVI. TBP, AMP, and AMX required AVI for activity (Fig. 1; see Fig. S1 in the supplemental material).

To confirm the results from the single-point screen, we determined the MICs of SUP, TBP, CXM, and AMX (the early-generation penicillin, AMP, was not followed up) with or without AVI (4 μg/mL). MIC was defined as 90% growth inhibition and derived from dose-response curves (29) (Table 1; Fig. S2). The MIC of SUP was 2.5 μM and AVI independent. TBP had an MIC of 4 μM in the presence of AVI. CXM showed a weak AVI dependency, with MICs of 5 μM and 10 μM with and without AVI, respectively. AMX exhibited a unique activity profile with a modest MIC of 25 μM in the presence of AVI but a substantially lower MIC_50_ of 3 μM, similar to the MIC_50_s of SUP, TBP, and CXM (Fig. S2). AVI alone had no growth-inhibitory activity (MIC > 100 μM). The two injectable comparators, IPM and FOX (which are both AVI independent [10]), showed MICs of 20 and 30 μM as reported (10). (Table 1; Fig. S2).

**TABLE 1:** Activity of SUP, TBP, CXM, and AMX without and with 4 μg/mL AVI against M. abscessus complex strains^*a*^

M. abscessus strain	*erm41*^*e*^ sequevar	CLR susceptibility	SUP		TBP		CXM		AMX		AVI^*b*^	IPM^*c*^	FOX^*c*^	CLR^*d*^
			MIC	MIC+	MIC	MIC+	MIC	MIC+	MIC	MIC+	MIC	MIC	MIC	MIC
Reference strains														
Subsp. *abscessus* ATCC 19977	T28	Resistant	2.5	2.0	25.0	4.0	10.0	5.0	>100	25.0^*a*^	>100	20.0	30.0	1.6
Subsp. *bolletii* CCUG50184T	T28	Resistant	3.5	2.0	30.0	5.0	20.0	10.0	>100	40.0	>100	30.0	30.0	5.0
Subsp. *massiliense* CCUG48898T	Deletion	Sensitive	7.0	5.0	40.0	7.0	30.0	10.0	>100	100.0	>100	40.0	45.0	0.4
Clinical isolates^*f*^														
Subsp. *abscessus* Bamboo	C28	Sensitive	3.0	2.5	25.0	4.0	10.0	8.0	>100	40.0	>100	20.0	35.0	0.4
Subsp. *abscessus* K21	C28	Sensitive	6.3	5.0	40.0	4.0	30.0	20.0	>100	100.0	>100	25.0	40.0	0.5
Subsp. *abscessus* M9	T28	Resistant	3.0	2.5	35.0	3.5	10.0	5.0	>100	40.0	>100	15.0	35.0	2.5
Subsp. *abscessus* M199	T28	Resistant	3.0	2.5	25.0	3.0	12.5	8.0	>100	75.0	>100	20.0	35.0	6.0
Subsp. *abscessus* M337	T28	Resistant	2.0	2.2	30.0	3.0	10.0	8.0	>100	60.0	>100	15.0	30.0	3.0
Subsp. *abscessus* M404	C28	Sensitive	3.5	2.5	30.0	3.5	20.0	7.0	>100	40.0	>100	20.0	35.0	0.4
Subsp. *abscessus* M422	T28	Resistant	2.5	2.0	25.0	3.0	10.0	5.0	>100	40.0	>100	12.5	35.0	1.5
Subsp. *bolletii* M232	T28	Resistant	2.5	2.0	40.0	3.5	15.0	8.0	>100	50.0	>100	15.0	40.0	2.0
Subsp. *bolletii* M506	C28	Sensitive	2.5	2.0	30.0	3.5	15.0	8.0	>100	70.0	>100	18.0	35.0	0.4
Subsp. *massiliense* M111	Deletion	Sensitive	4.0	3.5	35.0	4.0	15.0	10.0	>100	100.0	>100	30.0	35.0	0.4

a Cultures were treated with increasing concentrations of β-lactams without (MIC) or with 4 μg/mL AVI (MIC+) (28). Values present the concentrations (in micromolar) that achieved 90% inhibition of growth and are the means of three independent experiments. Note that AMX+AVI achieved 80% inhibition of growth at ~10 μM (see Fig. S2 in the supplemental material).

b AVI alone was included showing that the β-lactamase inhibitor did not achieve MIC up to 100 μM tested.

c The clinically used parenteral comparators IPM and FOX were only tested alone, as AVI does not affect activity of these β-lactams (Fig. 1) (10).

d CLR, assay control. Note increased MIC values for CLR-resistant strains.

e *erm41*, ribosome methylase gene conferring inducible CLR resistance. “C28” and “deletion” sequevars are inactive *erm41* alleles and susceptible to CLR. The “T28” sequevar is functional and confers inducible resistance to CLR (3).

f M. abscessus Bamboo (39), K21 (40), and M strains (41) were reported previously.

To confirm the growth inhibitory activity of SUP, TBP+AVI, CXM+AVI, and AMX+AVI in an orthogonal assay, we determined their MICs against M. abscessus ATCC 19977 by using the agar dilution method (30) and found agar MICs in the range of broth MICs (Fig. S3), with the exception of AMX, which, interestingly, had a lower agar than liquid MIC (6 μM versus 25 μM).

To determine whether the attractive activity of SUP, TBP+AVI, CXM+AVI, and AMX+AVI against the type strain M. abscessus subsp. *abscessus* ATCC 19977 was retained against the broader M. abscessus complex (31), broth MICs were measured against the reference strains of the two other subspecies, M. abscessus subsp. *bolletii* CCUG50184T and M. abscessus subsp. *massiliense* CCUG48898T, as well as a panel of clinical isolates, including *erm41* macrolide-resistant strains (Table 1; Fig. S2). Potency of the active β-lactams was largely comparable across the three subspecies of the M. abscessus complex. Again, CXM activity was 2- to 3-fold enhanced in the presence of AVI, and AMX+AVI displayed a modest MIC_90_ (Table 1) but substantially lower (3 to 4 μM) MIC_50_ (Fig. S2).

**FIG 2:**
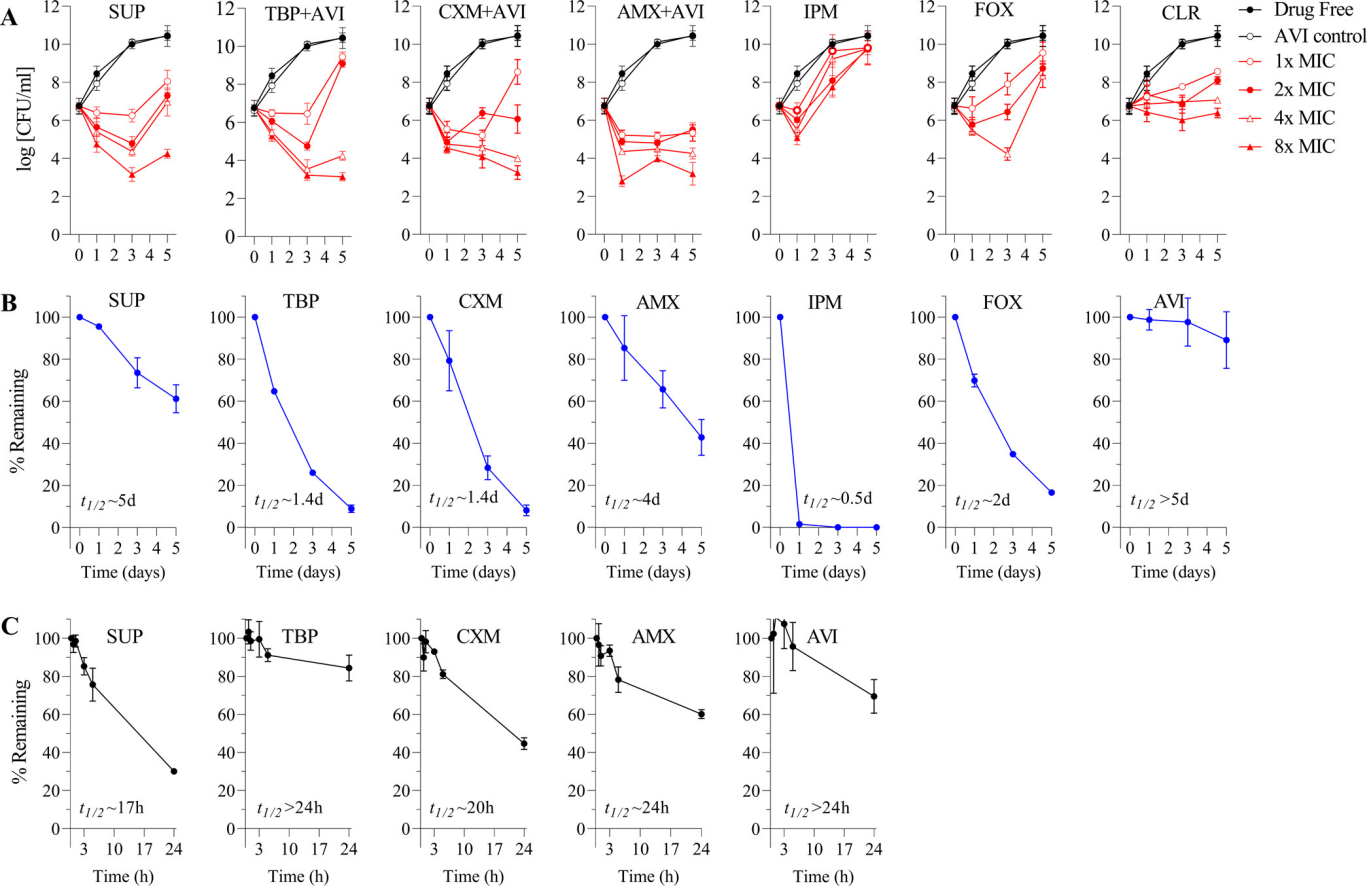
Dose-response time-kill curves of SUP, TBP+AVI, CXM+AVI, and AMX-AVI against M. abscessus ATCC 19977 and drug stability in culture medium and mouse plasma. (A) Time-concentration kill curves. Cultures of M. abscessus ATCC 19977 were treated with MIC (Table 1) and multiples of MICs of SUP (alone), TBP, CXM, and AMX (in combination with 4 μg/mL AVI) for 5 days, and viability of the cultures was monitored by CFU determination. CLR was included as assay control. IPM and FOX were included as clinically used parenteral comparators. (B) Stability of the β-lactams tested in panel A and AVI in 7H9 broth over a 5-day incubation period at 37°C. Percent remaining was calculated relative to time zero concentration (10 μM). Half-life was estimated from the decay curves. (C) Mouse plasma stability of the four oral β-lactams and AVI over a 1-day incubation period. Experiments in panel A were performed twice independently, generating similar data, and one representative set is shown. Experiments in panels B and C were carried out twice independently, and means and standard deviations are shown.

Next, we measured the bactericidal activity in dose-response time-kill experiments (29). M. abscessus ATCC 19977 cultures were grown in 7H9 and treated with MIC multiples of the β-lactams for 5 days (in the presence of 4 μg/mL AVI when required), and CFU were measured by plating samples on 7H10 agar. All four β-lactams achieved pronounced reductions in viable counts, up to 4-log CFU reduction after 3 days of incubation with 8-fold MIC (Fig. 2A). Interestingly, regrowth was observed in most cultures between days 3 and 5 for the oral β-lactams and even earlier for the injectables, which we hypothesized was associated with the limited aqueous stability of β-lactams (32, 33). To test the potential decay hypothesis, drug concentrations were followed in 7H9 medium over 5 days using high-pressure liquid chromatography coupled with tandem mass spectrometry (LC-MS/MS) (34). The lactam ring-containing drugs (i.e., all study drugs but not AVI) were unstable to various extents, mostly in line with the extent and timing of regrowth of the cultures (Fig. 2B). Half-lives (*t*_1/2_) of the β-lactams in 7H9 ranged from ~0.5 days for the markedly unstable IPM used as comparator (33) to ~5 days for the most stable SUP (Fig. 2B). Since β-lactams also undergo spontaneous and enzymatic hydrolysis in plasma (35), we measured stability of the four oral β-lactams and AVI in mouse plasma to determine which pair would be most suitable for *in vivo* efficacy in murine models of M. abscessus infection. We found that TBP, AMX, and AVI have a plasma *t*_1/2_ of ≥24 h (Fig. 2C), indicating that the TBP+AVI and AMX+AVI combinations could be prioritized as a case study to determine how these *in vitro* bactericidal synergies translate *in vivo*.

Taken together, these results confirm and extend prior studies showing attractive growth inhibitory and bactericidal anti-M. abscessus activity of SUP (36), TBP+AVI (28), CXM+AVI (10), and AMX+AVI (10), suggesting them as repurposing candidates.

To determine potential growth inhibition synergies of the four β-lactams, systematic pairwise checkerboard analyses were carried out with M. abscessus ATCC 19977 (37). AVI was included at 4 μg/mL in all assays, as at least one partner of each dual combination requires the β-lactamase inhibitor (Table 2). Interestingly, the three AMX-containing pairs were synergistic, while the other three were additive (Table 2). Synergistic activity of the AMX-containing pairs was confirmed in checkerboard assays against the broader M. abscessus complex and the clinical isolate collection. TBP and CXM, combined with AMX, retained strong synergistic activity against all tested strains and isolates, while SUP+AMX was additive against some of the isolates (Table 3).

**TABLE 2:** Checkerboard growth inhibition analysis of pairwise combinations of SUP, TBP, CXM, and AMX in the presence of 4 μg/mL AVI against M. abscessus ATCC 19977^*a*^

β-Lactam	MIC (μM)		FICI^*c*^	Interpretation^*d*^
	Alone^*e*^	Comb^*b*^		
SUP	4.0	0.5	0.61	Additive
TBP	2.5	1.2		
SUP	4.0	0.8	0.60	Additive
CXM	5.0	2.0		
SUP	4.0	0.5	0.37	Synergy
AMX	25.0	6.0		
TBP	2.5	1.5	0.80	Additive
CXM	5.0	1.0		
TBP	2.5	0.5	0.44	Synergy
AMX	25.0	6.0		
CXM	5.0	0.8	0.28	Synergy
AMX	25.0	3.0		

a The experiment was repeated once, yielding similar results.

b MIC of the combination (all in the presence of 4 μg/mL AVI, as at least one partner drug requires AVI for activity).

c Fractional inhibitory concentration index, calculated using the concentration at which at least 90% growth inhibition of the cultures was observed. FICI = (concentration of drug A in combination/concentration of drug A alone) + (concentration of drug B in combination/concentration of drug B alone).

d FICI, ≤0.5, synergistic; 0.5 to 1.0, additive; >1.0 to <2, indifferent; ≥2.0, antagonistic (42).

e MIC of single drugs, with 4 μg/mL AVI in the case of TBP, CXM, and AMX.

**TABLE 3:** Checkerboard growth inhibition analysis of SUP+AMX, TBP+AMX, and CXM+AMX in the presence of 4 μg/mL AVI against M. abscessus complex strains

M. abscessus strain	SUP+AMX					TBP+AMX					CXM+AMX				
	MIC (μM)^*a*^ of:				FICI^*b*^	MIC (μM)^*a*^ of:				FICI^*b*^	MIC (μM)^*a*^ of:				FICI^*b*^
	SUP alone	AMX alone	SUP comb	AMX comb		TBP alone	AMX alone	TBP comb	AMX comb		CXM alone	AMX alone	CXM comb	AMX comb	
Reference strains															
Subsp. *abscessus* ATCC 19977	2.5	25.0	0.5	7.0	0.48	4.0	25.0	0.4	7.0	0.38	6.3	25	0.5	4.0	0.24
Subsp. *bolletii* CCUG50184T	2.5	50	0.7	10.0	0.48	4.0	50.0	1.0	4.0	0.33	8.0	50.0	1.5	6.3	0.31
Subsp. *massiliense* CCUG48898T	4.0	100	1.0	25.0	0.50	8.0	100.0	1.0	10.0	0.22	12.5	100.0	1.5	25	0.37
Clinical isolates															
Subsp. *abscessus* Bamboo	2.5	50.0	0.5	10.0	0.40	4.5	40.0	0.5	5.0	0.24	8.0	50.0	1.5	5.0	0.29
Subsp. *abscessus* K21	5.0	100.0	1.0	25.0	0.45	5.0	100.0	1.5	12.5	0.43	12.5	100.0	1.5	6.3	0.18
Subsp. *abscessus* M9	2.5	50.0	0.5	10.0	0.40	4.0	50.0	0.8	6.3	0.33	6.3	50.0	0.8	12.5	0.38
Subsp. *abscessus* M199	2.5	100.0	0.5	25.0	0.45	4.5	80.0	0.5	10.0	0.24	8.0	100.0	1.5	10.0	0.29
Subsp. *abscessus* M337	2.0	75.0	0.4	25.0	0.53	4.5	75.0	0.8	12.5	0.34	8.0	75.0	1.5	10.0	0.32
Subsp. *abscessus* M404	2.0	50.0	0.4	12.5	0.45	4.5	50.0	0.8	6.3	0.30	8.0	50.0	1.0	5.0	0.23
Subsp. *abscessus* M422	2.5	50.0	0.5	10.0	0.40	4.5	50.0	0.5	6.0	0.23	8.0	50.0	1.5	5.0	0.29
Subsp. *bolletii* M232	2.5	50.0	0.8	12.5	0.57	4.5	50.0	1.0	6.3	0.35	8.0	50.0	2.0	6.3	0.38
Subsp. *bolletii* M506	2.5	75.0	0.5	15.0	0.40	4.5	75.0	0.5	10.0	0.24	8.0	80.0	1.5	10.0	0.31
Subsp. *massiliense* M111	2.5	100.0	0.8	25.0	0.57	4.5	75.0	0.8	12.5	0.34	8.0	100.0	1.5	8.0	0.27

a Alone and in combination as described in Table 2.

b FICI, ≤0.5, synergistic; 0.5 to 1.0, additive (42).

**FIG 3:**
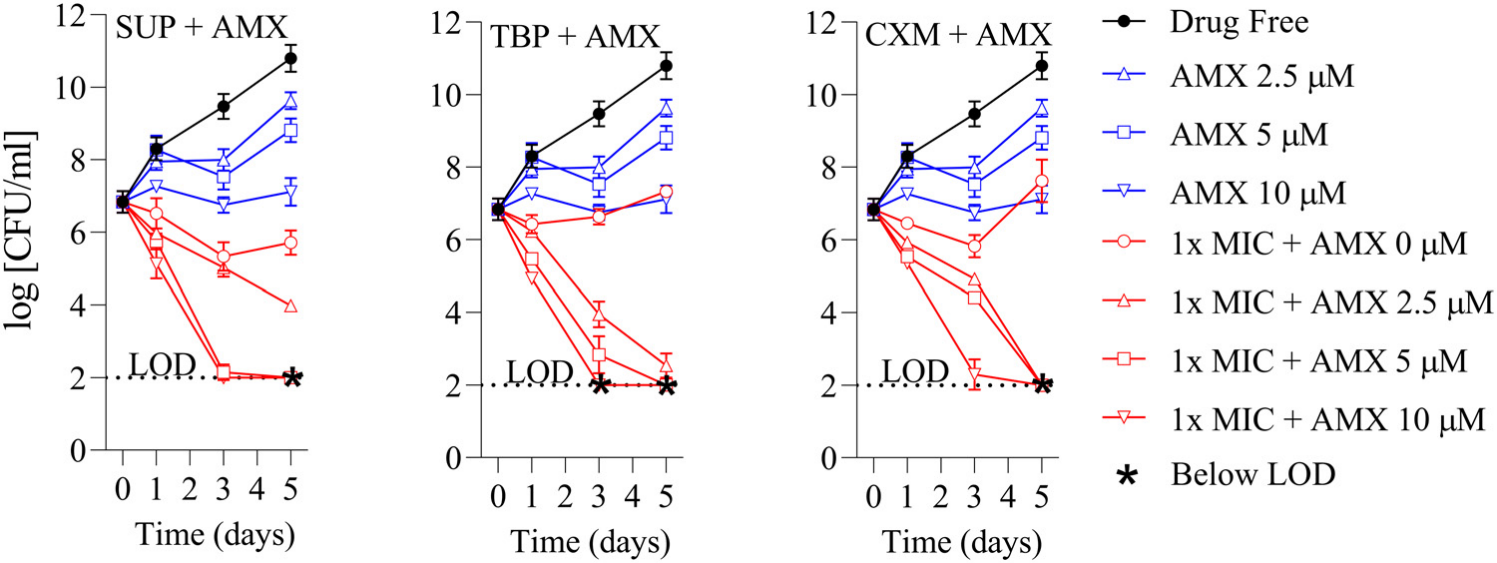
Time-kill curves of SUP+AMX, TBP+AMX, and CXM+AMX in the presence of 4 μg/mL AVI against M. abscessus ATCC 19977. Red curves represent cultures of M. abscessus ATCC 19977 that were treated with 1× MIC of SUP, TBP, or CXM (Table 1) with or without 10, 5, or 2.5 μM AMX in the presence of 4 μg/mL AVI for 5 days. Note, 10 μM AMX suppresses growth by 80% (Fig. S2). Bacterial viability was monitored by CFU determination. Blue curves: represent treatment of cultures with 10, 5, or 2.5 μM AMX alone in the presence of 4 μg/mL AVI. LOD, limit of detection (100 CFU/mL). The experiment was carried out twice independently, generating similar results, and one representative set of plots is shown.

To determine whether the three synergistic β-lactam pairs also exerted potentiation of bactericidal activity, time-kill experiments were carried out with M. abscessus ATCC 19977 in 7H9, and the effect of treatment on viability was measured by plating on 7H10 agar (29). To uncover potential bactericidal synergy, we combined SUP, TBP, or CXM at their MICs, concentrations that achieve little bactericidal effect (Fig. 2A), with AMX at 10 μM (at which the drug inhibits 80% growth [Fig. S2]) and 4 μg/mL AVI. Impressively, each of the three combinations achieved more than 4-log reduction in viable counts after 3 days of treatment (Fig. 3). In comparison, 8× MIC of each individual β-lactam was required to achieve a similar degree of killing (Fig. 2). Further reducing AMX concentration to 5 or 2.5 μM still achieved a 4-log reduction after 5 days of treatment, reinforcing the notion that AMX strongly potentiates the bactericidal activity of SUP, TBP, and CXM. In addition, the combinations not only killed effectively at lower concentrations than individual β lactams; they also prevented the regrowth observed in cultures treated with single drugs (Fig. 2A).

In conclusion, four oral β-lactams, SUP, TBP, CXM, and AMX, were identified as bactericidal against M. abscessus at clinically achievable concentrations. TBP, CXM, and AMX required the β-lactamase inhibitor AVI for optimal activity, whereas SUP’s activity was AVI independent. Pairwise combinations revealed three novel triple combinations (SUP or TBP or CXM with AMX plus AVI) showing both bacteriostatic and bactericidal synergy. Interestingly, all three β-lactam pairs contained AMX, which preferentially targets M. abscessus d,d-carboxypeptidase (15), whereas the carbapenem TBP was shown to inhibit l,d-transpeptidases Ldt_Mab1_ and Ldt_Mab2_ (14) and d,d-transpeptidases PonA1, PonA2, and PbpA (12). The specific targets of SUP and CXM have not been identified. However, similar to TBP, other penems and cephalosporins were also shown to preferentially target l,d- and d,d-transpeptidases (12–14). The differential inhibition of d,d-carboxypeptidase by AMX and of l,d- and d,d-transpeptidases by TBP, and possibly SUP and CXM, may provide the mechanistic basis for the observed synergistic effects of the AMX-containing β-lactam couples (38) since they would inhibit different enzymes of the same cellular process, i.e., peptidoglycan synthesis. In this context, it is interesting to note that AVI was shown to not only interact with the main M. abscessus β-lactamase but also with several l,d-transpeptidases as well as with d,d-carboxypeptidase (13). Oral forms of SUP and TBP, as well as AVI, are currently in clinical development for other diseases, and oral CXM and AMX are approved drugs (Table S1). Thus, the compounds and combinations identified in this study present drug candidates that can enter clinical development for M. abscessus lung disease. It is to note that the number of M. abscessus strains profiled in this study is relatively small. Follow-up studies with larger strain collections are required to confirm the results.
