# Supplementary material for: Strongly Bactericidal All-Oral β-Lactam Combinations for the Treatment of Mycobacterium abscessus Lung Disease
Source: Antimicrob Agents Chemother. 2022 Sep 1;66(9):e00790-22. doi: 10.1128/aac.00790-22 (PMC9487536; doi:10.1128/aac.00790-22)
Supplement: Supplemental file 1 — Supplemental material. Download aac.00790-22-s0001.pdf, PDF file, 0.5 MB [file aac.00790-22-s0001.pdf]

SUPPLEMENTAL MATERIALS

Strongly bactericidal all-oral β-lactam combinations for the treatment of *Mycobacterium abscessus* lung disease

Dereje A. Negatu,[a,b] Matthew D. Zimmerman,[a] Véronique Dartois,[a, c] Thomas Dick[a, c, d]#

[a] Center for Discovery and Innovation, Hackensack Meridian Health, Nutley, New Jersey, USA

[b] Center for Innovative Drug Development and Therapeutic Trials for Africa (CDT-Africa), Addis Ababa University, Addis Ababa, Ethiopia

[c] Department of Medical Sciences, Hackensack Meridian School of Medicine, Nutley, New Jersey, USA

[d] Department of Microbiology and Immunology, Georgetown University, Washington, DC, USA

Running Title: Combination of oral β lactams against *M. abscessus*

Keywords: Non-tuberculous mycobacteria, NTM, synergy, sulopenem, tebipenem, cefuroxime, amoxicillin, avibactam

#Address correspondence to Thomas Dick: thomas.dick.cdi@gmail.com

SUPPLEMENTAL TABLES

Table S1. Drugs used in the study: oral prodrug form, source, solvent and clinical status

No.	Drug	Prodrug form	Class	Catalog #	Source	Solvent	FDA status	Clinical development
1	Clarithromycin (CLR)	N/A	Macrolide	C9742	Sigma-Aldrich	DMSO	Approved	
2	Avibactam (AVI)	ARX-1796	Diazabicyclooctane	HY-14879A	MedChemExpress	DMSO	Approved[a]	[a] Phase 1 (NCT03931876)
3	Sulopenem (SUP)	Sulopenem etzadroxil	Penem	PZ0042	Sigma-Aldrich	DMSO	Not approved	[b] Phase 3 (NCT03357614)
4	Faropenem (FPM)	Faropenem medoxomil	Penem	F8182	Sigma-Aldrich	DMSO	Not approved	[c] Phase 2 (NCT02381470)
5	Tebipenem (TBP)	Tebipenem pivoxil	Carbapenem	161715-21-5	MuseChem	DMSO	Not approved	[d] Phase 3 (NCT03788967)
6	Imipenem (IPM)	N/A	Carbapenem	PHR1796	Sigma-Aldrich	Water	Approved	
7	Cephalexin (LEX)	N/A	Cephalosporin	PHR1848	Sigma-Aldrich	Water	Approved	
8	Cefaclor (CEC)	N/A	Cephalosporin	PHR1283	Sigma-Aldrich	Water	Approved	
9	Cefradine (CED)	N/A	Cephalosporin	C0690000	Sigma-Aldrich	DMSO	Approved	
10	Ceftibuten (CTB)	N/A	Cephalosporin	SML0037	Sigma-Aldrich	DMSO	Approved	
11	Cefprozil (CPR)	N/A	Cephalosporin	Y0001371	Sigma-Aldrich	DMSO	Approved	
12	Cefpodoxime (CPD)	Cefpodoxime proxetil	Cephalosporin	32344	Sigma-Aldrich	DMSO	Approved	
13	Cefixime (CFM)	N/A	Cephalosporin	CDS021590	Sigma-Aldrich	DMSO	Approved	
14	Cefdinir (CDR)	N/A	Cephalosporin	C7118	Sigma-Aldrich	DMSO	Approved	
15	Cefadroxil (CFR)	N/A	Cephalosporin	C0650000	Sigma-Aldrich	DMSO	Approved	
16	Cefuroxime (CXM)	Cefuroxime axetil	Cephalosporin	C4417	Sigma-Aldrich	DMSO	Approved	
17	Cefditoren (CDN)	Cefditoren pivoxil	Cephalosporin	HY-17452	MedChemExpress	DMSO	Approved	
18	Cefetamet (FET)	Cefetamet pivoxil	Cephalosporin	HY-B1894A	MedChemExpress	DMSO	Not approved	[e] Phase 4 (NCT04664803)
19	Cefoxitin (FOX)	N/A	Cephalosporin	C4786	Sigma-Aldrich	DMSO	Approved	
20	Penicillin V (PcV)	N/A	Penicillin	PHR2644	Sigma-Aldrich	DMSO	Approved	
21	Amdinocillin (AMD)	Pivmecillinam	Penicillin	32887-01-7	MuseChem	DMSO	Approved	
22	Flucloxacillin (FLX)	N/A	Penicillin	SML1023	Sigma-Aldrich	DMSO	Approved	
23	Dicloxacillin (DCX)	N/A	Penicillin	46182	Sigma-Aldrich	DMSO	Approved	
24	Cloxacillin (CLX)	N/A	Penicillin	PHR1922	Sigma-Aldrich	DMSO	Approved	
25	Ampicillin (AMP)	Bacampicillin	Penicillin	HY-B0522	MedChemExpress	Water	Approved	
26	Amoxicillin (AMX)	N/A	Penicillin	1031503	Sigma-Aldrich	DMSO	Approved	[f] Phase 2 (NCT02381470)

[a] Only the injectable form of AVI is approved. The oral AVI prodrug ARX-1796 is in Phase 1 clinical development. [b] Sulopenem etzadroxil, in clinical development for complicated urinary tract infections. [c] Faropenem, in clinical development for tuberculosis. [d] Tebipenem pivoxil hydrobromide, in clinical development for complicated urinary tract infections and acute pyelonephritis. [e] Cefetamet pivoxil, in clinical development for sinusitis. [f] Amoxicillin, in clinical development for tuberculosis.

SUPPLEMENTAL FIGURES

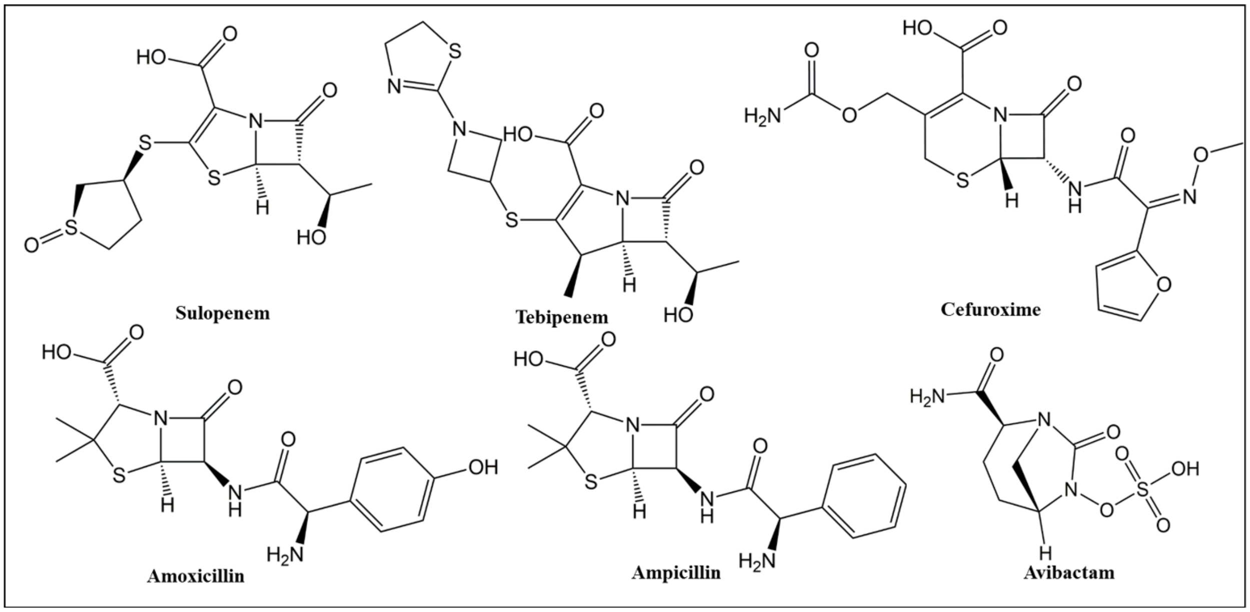

Fig. S1. Structures of β lactams SUP, TBP, CXM, AMX and AMP, and β-lactamase inhibitor AVI. Structures were derived from the PubChem database (https://pubchem.ncbi.nlm.nih.gov/) using the IUPAC name of the compounds.

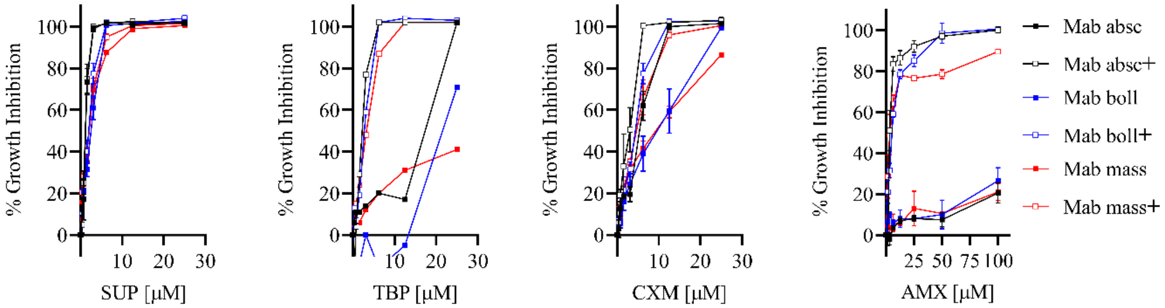

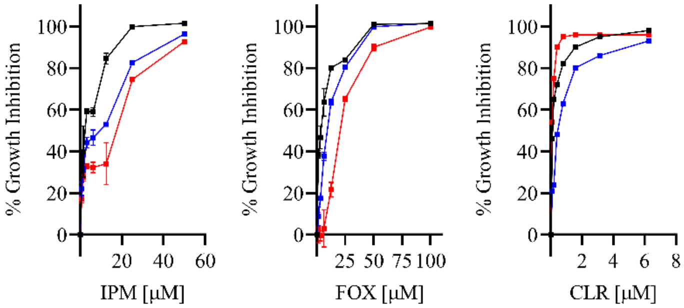

	MIC$_{50}$ [μM]						
	SUP	TBP	CXM	AMX	IPM	FOX	CLR
Mab absc	1.2	15.0	4.0	>100	2.0	3.5	0.12
Mab absc+	1.5	1.5	3.0	3.0	NA	NA	0.15
Mab boll	2.0	20.0	10.0	>100	12.0	8.0	0.50
Mab boll+	1.5	2.5	4.0	4.0	NA	NA	0.50
Mab mass	2.0	>25	10.0	>100	15.0	20.0	0.05
Mab mass+	1.5	3.0	4.0	3.5	NA	NA	0.05

Fig. S2. Growth inhibition dose-response curves for SUP, TBP, CXM and AMX with and without 4 ug/mL AVI against three *M. abscessus* subspecies reference strains. Mab absc, *M. abscessus* subsp. *abscessus* ATCC19977; Mab boll, *M. abscessus* subsp. *bolletii* CCUG50184T; Mab mass, *M. abscessus* subsp. *massiliense* CCUG48898T). '+', activity of β-lactam in the presence of 4 μg/mL AVI. CLR was included as assay control. IMP and FOX were included as clinically used parenteral comparators. The inserted table shows MIC$_{50}$ values (concentrations inhibiting 50% of growth) derived from the dose response curves. MIC values (concentrations inhibiting 90% of growth) derived from the curves are presented in Table 1. The experiments were carried out three time independently and means with standard deviations are shown.

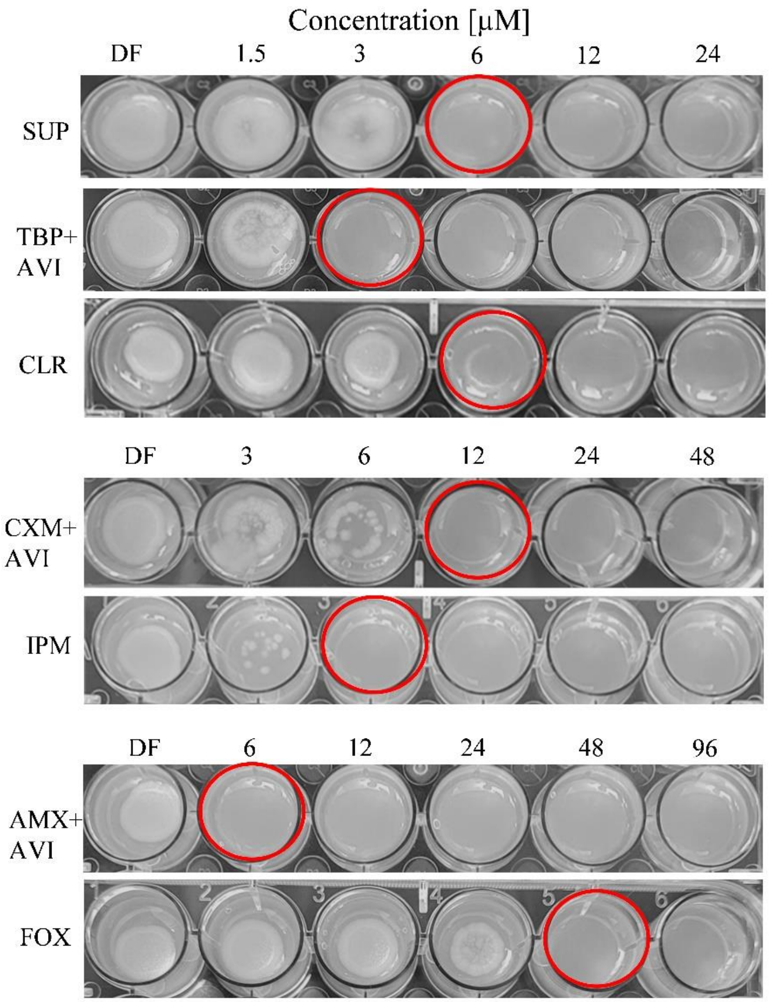

Fig. S3. Agar MIC of SUP, TBP+AVI, CXM+AVI and AMX+AVI for *M. abscessus* ATCC19977. 10^4 CFU *M. abscessus* ATCC19977 culture were spotted on agar containing increasing β-lactam concentrations as indicated. '+AVI', 4 µg/mL AVI was included in the agar. The agar MIC (first concentration preventing visible growth), indicated by red circles, were SUP, 6 µM (2.5 µM); TBP+AVI, 3 µM (4.0 µM); CXM+AVI, 12 µM (5 µM); AMX+AVI, 6 µM (25 µM). Agar MIC for IPM and FOX, included as comparators, were 6 µM (20 µM) and 48 µM (30 µM). Agar MIC for CLR, included as assay control, was 6 µM (1.6 µM). Numbers in parentheses show MICs determined in liquid cultures (Table 1). The experiment was carried out twice, yielding similar results.
